# Near-infrared nuclear markers for *Drosophila* imaging

**DOI:** 10.17912/micropub.biology.000639

**Published:** 2022-09-21

**Authors:** Yuanwang Pan, Cordelia Rauskolb, Kenneth D Irvine

**Affiliations:** 1 Waksman Institute, Rutgers University, 190 Frelinghuysen Rd, Piscataway NJ 08854 USA; 2 Laura and Isaac Perlmutter Cancer Center, New York University Langone Health, New York, New York, 10016, USA

## Abstract

Nuclear markers for live imaging are useful for counting and tracking cells, visualizing cell division, and examining the regulation of proteins that are controlled via entry or exit from the nucleus. Near-infrared fluorescent proteins have advantages over shorter wavelength fluorescent proteins, including reduced phototoxicity, less light scattering, and enabling multicolor live imaging. We have constructed and tested transgenic *Drosophila* expressing Histone H2Av iRFP fusion proteins, and confirmed that they can be used to label nuclei in both fixed and live tissue at multiple stages of development.

**Figure 1. His2Av:iRFP marks nuclei in Drosophila embryos and imaginal discs:**
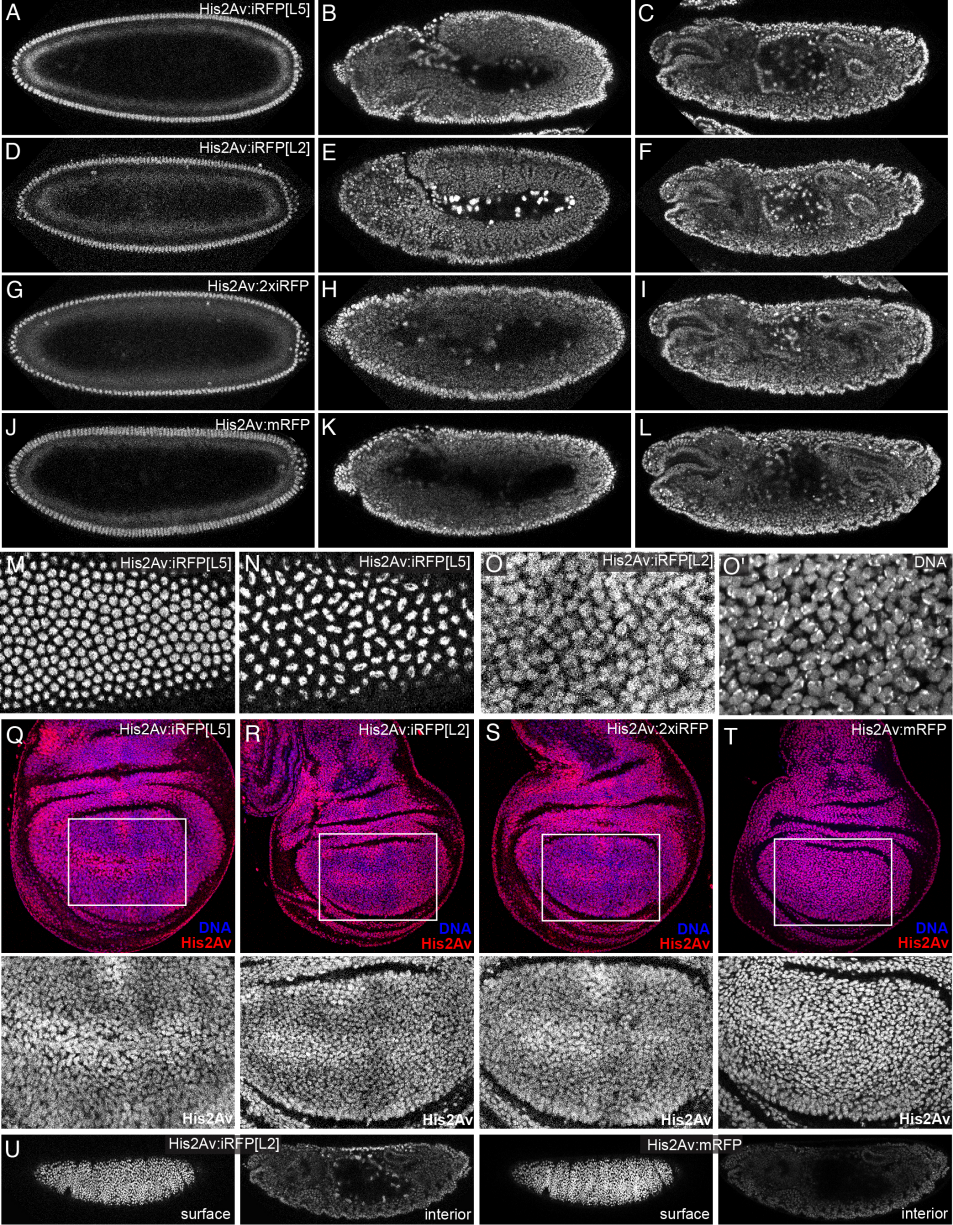
A-L) Live *Drosophila* embryos at blastoderm (A,D,G,J), extended germband (B,E,H,K) and dorsal closure (C,F,I,L) stages. A-C) His2Av:iRFP[L5]. D-F) His2Av:iRFP[L2]. G-I) His2Av:2xiRFP. J-L) His2Av:mRFP. M,N) close-ups of blastoderm embryos with His2Av:iRFP[L5] at interphase (M) and during nuclear divisions (N). O) Close-up of a fixed wing disc with His2Av:iRFP[L2] comparing His2Av:iRFP (left) with DNA stain (right, O’). Q-T) Fixed wing imaginal discs expressing tagged His2Av transgenes (red/white), stained for DNA using Hoechst33328 (blue): Q) His2Av:iRFP[L5], R) His2Av:iRFP[L2], S) His2Av:2xiRFP, T) His2Av:mRFP. White boxes identify regions for which His2Av is shown at higher magnification below. U) A live His2Av:iRFP[L2]/ His2Av:mRFP *Drosophila* embryo at dorsal closure stage. Imaging conditions where the intensities of the iRFP and RFP signals appear similar at the surface were established, and then used to image the same embryo at depth (interior image shown is 37 µm deeper than the surface view).

## Description

**Introduction**

The discovery and engineering of fluorescent proteins has revolutionized many areas of biology (Rodriguez et al., 2017). Widely used applications include labelling proteins or cellular structures to monitor post-translational regulation, cellular organization, and cell and protein dynamics, particularly for live imaging approaches. Analysis of labelled proteins often benefits from comparisons to independent markers, such as fluorescent proteins that label cell structures like membranes or nuclei. Nuclear markers are particularly useful for counting and tracking cells, due to the separation of nuclei between neighboring cells. Nuclear markers are also useful for visualizing cell division, and for examining the regulation of proteins that are controlled via entry or exit from the nucleus, including transcription factors like Yorkie (Oh and Irvine, 2010).

A wide range of fluorescent proteins have been created, varying in their excitation and emission wavelengths, brightness, and photostability (Rodriguez *et al.* , 2017). While proteins with excitation and emission characteristics similar to those of GFP and RFP are popular, far-red, or near-infrared fluorescent proteins including IFP1.4 and iRFP have some advantages over shorter wavelength fluorescent proteins. Longer excitation wavelengths are associated with reduced phototoxicity (Icha et al., 2017), making near-infrared fluorescent proteins particularly useful for live imaging experiments. Near infrared wavelengths are subject to less light scattering than visible or ultraviolet wavelengths in biological tissues, and so can penetrate deeper into biological samples (Filonov et al., 2011; Ntziachristos, 2010). Additionally, using near-infrared fluorescent proteins as cellular markers keeps other wavelengths available to tag proteins of interest, facilitating multicolor live imaging experiments.

Here we describe the creation and characterization of transgenic *Drosophila* expressing a Histone H2 protein tagged with the near-infrared fluorescent protein iRFP (Filonov *et al.* , 2011). His2Av:iRFP marks nuclei in both embryos and imaginal discs, and can be imaged both live and in fixed tissue. It should be broadly useful, particularly for live imaging studies in which it is desirable to label nuclei.

**Results**

**Generation of His2Av:iRFP-expressing flies**

To construct a *Drosophila* near-infrared nuclear marker, we fused Histone H2A variant (His2Av, CG5499) with the near-infrared fluorescent protein iRFP. His2Av was chosen as it has been successfully used previously for fusions with GFP and RFP (Clarkson and Saint, 1999; Schuh et al., 2007). iRFP, a phytochrome-based fluorescent protein with excitation and emission maxima at 690 nm and 713 nm, respectively, was chosen as a relatively bright and photostable near-infrared fluorescent protein (Filonov *et al.* , 2011). iRFP was fused to His2Av within a genomic plasmid, using a plasmid that contained a genomic His2Av:mRFP fusion construct as a starting point (pCaSpeR4-gHis2AvD-mRFP1)(Schuh *et al.* , 2007). This plasmid includes genomic DNA from His2Av, such that His2Av is expressed under its own promoter. iRFP was isolated from the expression plasmid pShuttle-CMV-iRFP (Filonov *et al.* , 2011) by PCR amplification, and then subcloned into plasmid pCaSpeR4-gHis2AvD-mRFP1 that had been digested to excise mRFP, resulting in the plasmid pCaSpeR4-gHis2AvD-iRFP.

pCaSpeR4-gHis2AvD-iRFP was co-injected with transposase helper plasmid into *w ^1118^ Drosophila* to obtain transgenics via P-element mediated transformation. Several transgenic lines were identified, and two with robust expression were kept: L5, on the 2 ^nd^ chromosome and L2, on the 3 ^rd^ chromosome. Additional insertions that were obtained had weaker or mosaic expression, and they were discarded. In an attempt to see if even brighter near-infrared nuclear markers could be generated, we also created a His2Av fusion construct with two tandemly repeated copies of iRFP (His2Av:2x-iRFP) and obtained an insertion of this transgene on the X chromosome. The His2Av:iRFP and His2Av:2x-iRFP transgenes did not have any noticeable effects on viability, fertility, or morphology of transgenic flies.

**Expression of His2Av:iRFP transgenes in embryos and imaginal discs**

Expression of each of the His2Av:iRFP and His2Av:2x-iRFP transgenes was examined in homozygous live *Drosophila* embryos by confocal microscopy. They were detected at similar levels and appear to be expressed in all nuclei throughout embryonic development (Fig. A-I). Expression of the His2Av:2xiRFP transgene appeared similar to the transgenes with only one copy of iRFP. For comparison, we also examined embryos expressing a His2Av:mRFP transgene (Schuh *et al.* , 2007) (Fig. J-L). The expression pattern and ease of detection of His2Av:iRFP and His2Av:mRFP were similar. Nuclear His2Av:iRFP signals could be detected in internal tissues in the middle of the embryo, but nuclei nearer the surface were more readily detected. iRFP-labelled histones were also suitable for detecting nuclear divisions in live embryos (Fig. M,N).

His2Av:iRFP and His2Av:2xiRFP were also detected in the nuclei of wing imaginal discs (Fig. Q-S). Some regions of the wing disc exhibited higher expression levels, including nuclei near the dorsal-ventral compartment boundary. To further investigate this, the pattern of His2Av:iRFP was compared to other nuclear markers within the same tissue by staining with the DNA dye Hoechst 33342, and by examining the pattern of His2Av:mRFP expression. This confirmed that while the His2Av:iRFP and His2Av:2xiRFP transgenes appear to be expressed in all nuclei, the levels of expression between different regions of the wing disc are uneven. This contrasted with the expression detected from the His2Av:mRFP transgene, which appeared similar to the pattern detected by Hoechst 33342 (Fig. T). We also note that within a single nucleus, both His2Av:iRFP and His2Av:mRFP fusions have more uniform staining than Hoechst 33342 (Fig. O).

To directly compare the relative ability to image His2Av:iRFP versus His2Av:mRFP at depth, we crossed flies expressing these transgenes together, such that each transgene could be imaged simultaneously in the same embryo under the same conditions. In live embryos heterozygous for His2Av:iRFP and His2Av:mRFP, we established imaging conditions where the signal intensities from each transgene at the surface appeared similar. We then used these same imaging conditions to section through embryos on a confocal microscope. At depth (eg, ~30-40 µm below the surface), the His2Av:iRFP transgene generated noticeably better signals than His2Av:mRFP (Fig. U).

**Conclusions**

Our results establish His2Av:iRFP and His2Av:2x-iRFP transgenes as near-infrared nuclear markers for *Drosophila* imaging. While they can be used with fixed tissue, we anticipate that they will be most useful for live imaging experiments, particularly in cases where other proteins are labeled with fluorescent proteins like GFP and RFP, or when imaging deep below the surface.

The observation of an expression pattern in wing discs with the His2Av:iRFP transgenes was unexpected, as expression is driven by the endogenous promoter in a genomic construct, and we used the same construct as was used to create the His2Av:mRFP transgene, which doesn’t show this pattern. Moreover, since a similar pattern is observed with three independent insertions, it is unlikely to be a position effect. We infer that there may be some sequences within the iRFP gene itself that modify the expression pattern in wing discs. The possibility that expression is uneven could be a factor in experiments where it is desirable to normalize the nuclear signal of a protein of interest to a neutral nuclear marker. Nonetheless, the His2Av:iRFP and His2Av:2x-iRFP transgenes are broadly expressed and so should still be useful for a variety of live imaging experiments.

## Methods

**Plasmid construction and transformation**

To create pCaSpeR4-gHis2AvD-iRFP, pShuttle-CMV-iRFP (Filonov *et al.* , 2011) was obtained from Addgene (#31856). iRFP was isolated from pShuttle-CMV-iRFP using primers iRFP-F2 and iRFP-R2. The sequence of iRFP-F2 is 5’ ttctgtcgcaggcc **tac** gga **atg** gcggaaggatccgtcg 3’, where the underlined nucleotides are homologous to sequences in the destination vector and the nucleotides downstream of this are homologous to iRFP, and the triplets highlighted in bold correspond to the last amino acid of His2Av (tac) and first amino acid of iRFP (atg). The sequence of iRFP-R2 is 3’ gatcggcgtgatggaagagtga gaagcttaagccagtcggca 5’, where the underlined nucleotides are homologous to iRFP and the nucleotides downstream of this are homologous to the 3’ non-coding region of His2Av. The downstream portion of His2Av was obtained from plasmid pCaSpeR4-gHis2AvD-mRFP1, using primers His2AvD down F1 and His2AvD down R2. The sequence of His2AvD down F1 is 5’ gaagcttaagccagtcggcaatc 3’, and the sequence of His2AvD down R2 is 3’ agagtcgacctcgaacgttaacg 5’. The plasmid pCaSpeR4-gHis2AvD-mRFP1 was digested with AgeI and XbaI to remove mRFP, and the two PCR products were inserted into the plasmid using Gibson assembly.

To create plasmid pCaSpeR4-gHis2AvD-2xiRFP, we used primers 2xiRFP-F2 and 2xiRFP-R2 **.** The sequence of 2xiRFP-F2 is 5’ ttctgtcgcaggcc **tac** gga ttcgttaacagatct **atg** gcgga 3’, where the underlined nucleotides are homologous to sequences in the destination vector and the nucleotides downstream of this are homologous to vector and iRFP sequences from the plasmid pUAST-2xiRFP and the triplets highlighted in bold correspond to the last amino acid of His2Av (tac) and first amino acid of iRFP (atg). The sequence of 2xiRFP-R2 is 3’ gaagagtgagtcgacgcgg gaagcttaagccagtcggca 5’where the underlined nucleotides are homologous to vector and 2xiRFP and the nucleotides downstream of this are homologous to the 3’ non-coding region of His2Av.

Plasmids were transformed into *Drosophila* using a commercial service (Bestgene). Transformation was done into *w ^1118^* , using the transposase-expressing helper plasmid pUChsπ∆2-3. The pCaSpeR4 vector includes a *white* gene. Insertions of His2Av:iRFP were obtained on the second chromosome (L5) and on the third chromosome (L2). An insertion of His2Av:2xiRFP was obtained on the X chromosome.

**Histology and Imaging**

Except where noted (Fig. U), all imaging was done on animals homozygous for the His2Av transgenes, and the transgenes were maintained as homozygous stocks. Embryos were collected on apple juice agar plates with yeast at 25°C, dechorionated with bleach and rinsed with water. They were then transferred to an apple juice agar plate to select embryos of the desired ages. A coverslip was adhered via double-sided tape to a metal Leica FrameSlide with the polyethylene terephthalate membrane removed. The embryos were transferred to the coverslip in halocarbon oil 27 (Sigma) and then covered with a YSI permeable membrane. Wing imaginal discs were dissected and fixed as described previously (Rauskolb and Irvine, 2019). DNA was stained using Hoechst 33342 (Life Technologies). Confocal images were captured on a Leica SP8 confocal microscope. iRFP was excited at 670nm, as this was the longest excitation wavelength available on our system. While this is slightly below the peak excitation wavelength for iRFP (690 nm) (Filonov *et al.* , 2011), it was adequate for visualizing His2Av:iRFP.
